# Treatment of latent tuberculosis in a child with mucopolysaccharidosis type I receiving enzyme replacement therapy: A case report

**DOI:** 10.3389/fped.2022.973193

**Published:** 2022-08-16

**Authors:** Lauma Vasilevska, Madara Auzenbaha, Ieva Grinfelde, Anita Skangale

**Affiliations:** ^1^Clinic of Medical Genetics and Prenatal Diagnostics, Children's Clinical University Hospital, Riga, Latvia; ^2^Department of Biology and Microbiology, Riga Stradiņš University, Riga, Latvia; ^3^Riga Outpatient Department, Centre of Tuberculosis and Lung Diseases, Riga East University Hospital, Riga, Latvia

**Keywords:** enzyme replacement therapy (ERT), mucopolysaccharidosis type I, laronidase, multi drug resistant tuberculosis (MDR-TB), latent tuberculosis treatment

## Abstract

Mucopolysaccharidosis type I S (MPS IS) is a rare autosomal recessive lysosomal storage disorder caused by mutations in the *IDUA* gene, leading to a deficiency of the enzyme alpha-L-iduronidase. Enzyme replacement therapy (ERT) reduces lysosomal storage in the liver and improves clinical manifestations. To date, there are no published reports of tuberculosis (TB) treatment in MPS IS patients receiving ERT and as such it is not known whether both conditions can be treated simultaneously. Here, we report the case of a 14-year-old male with MPS IS receiving ERT with laronidase who was diagnosed with a latent TB infection after being in contact with a multi-drug-resistant TB patient. He received prophylactic TB treatment with moxifloxacin for 6 months. No complications were reported and there has been no active TB disease. Our case report demonstrates that TB and MPS IS can be treated simultaneously without serious adverse effects.

## Introduction

Mucopolysaccharidosis type I S (MPS IS) or Scheie syndrome (OMIM #607016) is a rare autosomal recessive lysosomal storage disorder caused by a deficiency of the enzyme alpha-L-iduronidase (EC 3.2.1.76), leading to storage of dermatan sulfate and heparan sulfate (1). MPS IS is caused by mutations in the *IDUA* gene, located on chromosome 4p16.3 (2). In MPS IS patients, treatment with recombinant human alpha-L-iduronidase (laronidase) – the deficient enzyme – reduces lysosomal storage in the liver and improves some clinical manifestations whilst stabilizing others (3). Although the incidence of tuberculosis (TB) in Latvia is decreasing (23 cases per 100,000 in 2020), Latvia remains in the top six EU and European Economic Area countries with the highest TB case notifications and there is a high burden of multi-drug resistant (MDR) TB (4, 5). BCG vaccination in Latvia is offered to all newborns between the 2nd and 5th day after birth; booster vaccinations are not given at an older age (6). Routine screening for TB is not performed; however, patients (including children) are tested if they show signs/symptoms or have been in contact with a TB patient (7). To date, there are no published reports of TB treatment in MPS IS patients receiving enzyme replacement therapy (ERT) and as such it is not known whether both conditions can be treated simultaneously. Here, we report the case of a 14-year-old male with MPS IS receiving ERT who was diagnosed with a latent TB infection (positive IGRA test) after contact with an MDR TB patient.

## History and presentation

The patient was born at 40 weeks' gestation to non-consanguineous parents and received his BCG vaccination post birth. Early psychomotor development was normal. During preschool years, he suffered from frequent upper respiratory tract infections, sinusitis, otitis media and bronchial asthma. At the age of 5 years, the patient developed knee pain and joint stiffness. At the age of seven years, he was diagnosed with polyarthritis and chronic uveitis and was initially treated for juvenile idiopathic arthritis. However, when an elevated level of mucopolysaccharide in his urine was detected, further diagnostic enzyme analysis revealed pathologically low alpha-L-iduronidase activity and two heterozygous disease-causing mutations in the *IDUA* gene were identified – the first one in exon 2 (c.208C>T p.Q70^*^) and the second one in intron 12 (c.1727+4C>T). A diagnosis of MPS type I was made, and he was put under the supervision of a multidisciplinary team. Consequently, ERT was started with intravenous laronidase 100 U/kg once a week. In response, the patient's joint mobility improved, and no joint pain was reported. However, he continued to endure mild bilateral carpal tunnel syndrome, extension contractures of the distal phalanges, restricted mobility of the wrists and rigid equinus deformity of both feet. The lattermost was corrected by reconstructive surgeries and bilateral Achilles tendon extension. The patient developed mild mitral regurgitation, but this did not require any treatment. His lung function (FVC, FEV1) also improved and fewer respiratory infections were noted. The patient wears spectacles. His most recent ophthalmologic evaluation in 2022 revealed disseminated point-like dystrophic changes in the cornea (corneal dystrophy) and hypermetropic astigmatism with amblyopia ex anopsia. He has never displayed a neurological or intellectual deficit and attends a mainstream school.

In 2017, at the age of 10 years, the patient was in contact with a family member with MDR TB resistant to the first line of anti-tuberculosis drugs. He was subsequently tested for TB infection (Mantoux tuberculin skin test) and a skin induration of 14 mm was observed, but IGRA test was negative. A lung CT scan was conducted, and no pathologic changes were detected. A diagnosis of latent tuberculosis infection was made, but during this period, prophylaxis with second line anti-tuberculosis drugs was not recommended by the WHO, so the prophylaxis was not prescribed. No other family members were infected.

In 2021, at the age of 14 years, the patient was in contact with a close neighbor with MDR TB (resistant to isoniazid, rifampicin and pyrazinamide; preserved sensitivity to moxifloxacin). All family members were tested again, and the patient showed a positive IGRA test. Lung and intrathoracic lymph node CT scans were conducted, and no TB process was detected. A diagnosis of latent TB infection was made and prophylactic treatment was started with peroral moxifloxacin 400 mg once a day for 6 months. Due to the high burden of MDR TB in Latvia and to ensure patient compliance, monitoring of TB drug treatment via Skype (DOT or directly observed therapy) is used with patients who cannot attend a medical institution each day to receive the prescribed medication. DOT was used in this case.

The patient reported nausea and dizziness shortly after administration of moxifloxacin and vomiting once a week, which continued throughout the duration of the treatment. During the treatment, no abnormal liver function tests were observed, the corrected QT interval on ECG was normal, and the patient continued to receive ERT once a week. No complications were reported and no active TB disease has been detected to date.

## Discussion

People with a latent TB infection do not feel ill and do not have any symptoms. They are infected with *Mycobacterium tuberculosis*, but do not have active TB disease. The only sign of TB infection is a positive reaction to the tuberculin skin test or IGRA test (8). Most children identified with a latent TB infection have been infected relatively recently compared with adults who may have been infected decades previously. Children and adolescents are at higher risk for progression from infection to active TB disease (with the potential for disseminated disease) than adults (9, 10).

Since optimal prophylactic therapy for MDR TB infection has not been established, it is individualized based on the drug resistance pattern of the mycobacteria from the source of infection. It was unclear which of the two MDR TB contacts had infected the patient, but both sources had resistance to first-line drugs (isoniazid, rifampicin and pyrazinamide) with preserved sensitivity to moxifloxacin. Considering that WHO recommends prophylaxis with fluoroquinolone drugs in case of MDR MBT, a second line treatment with moxifloxacin was chosen and it was verified that there were no known drug interactions with laronidase (the patient's ERT) reported in the literature (11). Additionally, moxifloxacin is less hepatotoxic than levofloxacin, which is the more commonly used second-line drug (12, 13). The patient's liver function was monitored throughout his 6-month treatment. Of note, prophylactic second line treatment for TB in Latvia has been available for only the past 2 years.

Interestingly, the patient was the only member of his family to become infected with TB, despite having the least exposure to the MDR TB-infected contacts. Lysosomal storage diseases (such as MPS IS) reduce macrophage endocytic recycling and impair migration to newly infecting mycobacteria (14). This incapacitation of highly microbicidal first-responding macrophages may contribute to TB susceptibility (14).

This case report demonstrates that TB and MPS IS can be successfully treated at the same time with no serious adverse effects.

## Data availability statement

The original contributions presented in the study are included in the article/supplementary material, further inquiries can be directed to the corresponding author.

## Author contributions

IG carried out the genetic diagnostics. AS was responsible for patient's diagnostics and treatment. MK was responsible for patient's treatment and supervised the writing process of the article. LV wrote the initial manuscript, led revisions, and submitted the manuscript. All authors have been involved in the conception and design of the article, revising it critically for important intellectual content, read and approved the final manuscript.

## Conflict of interest

The authors declare that the research was conducted in the absence of any commercial or financial relationships that could be construed as a potential conflict of interest.

## Publisher's note

All claims expressed in this article are solely those of the authors and do not necessarily represent those of their affiliated organizations, or those of the publisher, the editors and the reviewers. Any product that may be evaluated in this article, or claim that may be made by its manufacturer, is not guaranteed or endorsed by the publisher.

## References

[1] Kubaski F de Oliveira Poswar F Michelin-Tirelli K Matte UDS Horovitz DD Barth AL. Mucopolysaccharidosis type I. Diagnostics. (2020) 10:161. 10.3390/diagnostics10030161 32188113 PMC7151028

[2] Scott HS Ashton LJ Eyre HJ Baker E Brooks DA Callen DF. Chromosomal localization of the human alpha-L-iduronidase gene (IDUA) to 4p16.3. Am J Hum Genet. (1990) 47:802–7. 2220820 PMC1683689

[3] Hahn S. Mucopolysaccharidoses: treatment - UpToDate. (2021). Available online at: https://www.uptodate.com/contents/mucopolysaccharidoses-treatment?search=mucopolysaccharidosis&source=search_result&selectedTitle=2~65&usage_type=default&display_rank=2#H3977465703 (accessed April 12, 2022).

[4] World Health Organization Global Tuberculosis Report. Incidence of tuberculosis (per 100,000 people) - Latvia | Data. (2020). Available online at: https://data.worldbank.org/indicator/SH.TBS.INCD?locations=LV (accessed April 11, 2022).

[5] Balakrishnan VS. Managing tuberculosis in the Baltic States. Lancet Respir Med. (2019) 7:653–4. 10.1016/S2213-2600(19)30219-X 31221567

[6] European Centre for Disease Prevention Control (ECDC). Vaccine Scheduler | ECDC. (2022). Available online at: https://vaccine-schedule.ecdc.europa.eu/Scheduler/ByDisease?SelectedDiseaseId=14&SelectedCountryIdByDisease=-1 (accessed April 11, 2022).

[7] Latvia: migrant health guide - GOV.UK. Updated 30 May 2019. (2014). Available online at: https://www.gov.uk/guidance/latvia-migrant-health-guide (accessed April 13, 2022).

[8] Division Division of Tuberculosis Elimination V. H. National Center for HIV, STD and TB Prevention, and Centers for Disease Control and Prevention. Fact Sheets | General | Latent TB Infection vs. TB Disease | TB | CDC. (2014). Available online at: https://www.cdc.gov/tb/publications/factsheets/general/ltbiandactivetb.htm (accessed April 11, 2022).

[9] Nolt D Starke JR. Tuberculosis infection in children and adolescents: testing and treatment. Pediatrics. (2021) 148:e2021054663. 10.1542/peds.2021-054663 34851422

[10] Sterling TR Njie G Zenner D Cohn DL Reves R Ahmed A. Guidelines for the treatment of latent tuberculosis infection: recommendations from the national tuberculosis controllers association and CDC, 2020. MMWR Recomm Rep. (2020) 69:1–11. 10.15585/mmwr.rr6901a1 32053584 PMC7041302

[11] “Drug, Interaction https://www.Report-Drugs.com” Available online at: https://www.drugs.com/interactions-check.php?drug_list=1437-10838,1659-0 (accessed April 13, 2022).

[12] Levofloxacin. LiverTox: Clinical and Research Information on Drug-Induced Liver Injury. (2020). Available online at: https://www.ncbi.nlm.nih.gov/books/NBK548357/ (accessed July 26, 2022).

[13] Moxifloxacin. LiverTox: Clinical and Research Information on Drug-Induced Liver Injury. (2020). Available online at: https://www.ncbi.nlm.nih.gov/books/NBK548166/ (accessed August 6, 2022).

[14] Berg RD Levitte S O'Sullivan MP O'Leary SM Cambier CJ Cameron J. Lysosomal disorders drive susceptibility to tuberculosis by compromising macrophage migration. Cell. (2016) 165:139–52. 10.1016/j.cell.2016.02.034 27015311 PMC4819607

